# From inflammation to neurodegeneration: an exploratory pilot study of a diagnostic framework for progression in MS

**DOI:** 10.3389/fneur.2026.1767921

**Published:** 2026-05-20

**Authors:** Tobias Hegelmaier, Khaldoon O. Al-Nosairy, Alexander Duscha, Antonia Lipp, Henrike Marie Nowitzki, Christiane Desel, Shahram Taherzadeh Amlashi, Léon Beyer, Klaus Gerwert, Hagen Thieme, Michael B. Hoffmann, Aiden Haghikia

**Affiliations:** 1 Department of Neurology and Clinical Neurophysiology, Hannover Medical School, Hannover, Germany; 2 Department of Neurology, University Hospital Magdeburg, Magdeburg, Germany; 3 Department of Ophthalmology, University Hospital Magdeburg, Magdeburg, Germany; 4 Department of Biophysics, Ruhr-University Bochum, Bochum, Germany; 5 Center for Protein Diagnostics (ProDi), Biospectroscopy, Ruhr-University Bochum, Bochum, Germany; 6 Center for Behavioral Brain Sciences, Magdeburg, Germany

**Keywords:** biomarkers, computational sciences, multiple sclerosis, neuroimmunology, ophthalmology, progression markers

## Abstract

**Background:**

The pathogenesis of Multiple Sclerosis (MS) involves a dynamic interplay between (auto-)inflammation and neurodegeneration, driving its relapsing and progressive course. While both processes have been shown to be involved throughout disease with different emphasis, relapsing MS (RMS) is characterized by acute inflammatory activity, and secondary progressive MS (SPMS) involves chronic, diffuse neurodegeneration. Identifying biomarkers to predict progression independent of relapse activity (PIRA) is critical for improving diagnostic accuracy and treatment strategies. This study aimed to integrate inflammatory and neurodegenerative biomarkers into a Classification And Regression Tree (CART) model to distinguish RMS from SPMS.

**Methods:**

We employed a multimodal approach by combining functional as well as structural retinal assessment via multifocal electroretinography (mfERG) and optical coherence tomography (OCT), serum neurofilament light chain (sNfL) and glial fibrillary acidic protein (GFAP) levels via multiplex technology, and subsequent deep immunophenotyping. A CART model was constructed and trained to classify people with MS (PwMS) into RMS or SPMS categories based on these biomarkers.

**Results:**

Our results revealed significant thinning of the retinal nerve fiber layer (RNFL) and ganglion cell-inner plexiform layer (GCIPL) in pwMS, with more pronounced reductions in SPMS, while functional mfERG-readouts did not differ between MS subgroups. Neurodegenerative markers sNfL and GFAP were elevated in pwMS compared to healthy controls, with higher levels in SPMS. Immunophenotyping showed increased levels of non-classic and intermediate monocytes in SPMS. The CART and Random Forest models identified sNfL, GCIPL thickness, and frequency of intermediate monocytes as the most accurate predictors, achieving approximately 80% accuracy in distinguishing RMS from SPMS.

**Discussion:**

These findings suggest that combining sNfL, GCIPL thickness, and monocyte subsets provides an experimental, but robust diagnostic framework for differentiating RMS from SPMS. This approach could enable earlier identification of disease progression, facilitating tailored therapeutic interventions. Future studies should validate this model in larger cohorts to enhance its clinical applicability.

## Introduction

The pathogenesis of Multiple Sclerosis (MS) is a complex interplay between immune mechanisms and neurodegeneration, which together drive the relapsing and progressive courses of the disease. Adaptive immune responses, mediated by T- and B-lymphocytes, are central to the formation of acute focal lesions and clinical relapses. In contrast, innate immune processes contribute to a more diffuse, chronic inflammation and neurodegeneration, often referred to as “smoldering MS,” which is believed to be the underlying process of disease progression. This chronic pathology is less responsive to conventional therapies that target acute inflammatory activity, underscoring the urgent need for biomarkers that can reliably predict progression independent of relapse activity (PIRA) (1, 2).

The lack of validated biomarkers for PIRA in MS remains a critical gap in clinical practice. While high-efficacy treatments such as B-cell-depleting therapies effectively suppress focal lesion formation, their ability to prevent long-term disability remains limited. This therapeutic gap highlights the importance of identifying markers that can capture the diffuse neurodegenerative processes driving progression. For instance, serum neurofilament light chain (sNfL) has emerged as a marker of neuronal injury and therapy response in active disease. However, its utility in predicting PIRA remains unclear. Similarly, glial fibrillary acidic protein (GFAP) has shown promise as a biomarker for progression due to its association with astrocytic activation and brain-wide neurodegeneration (1, 3, 4).

Furthermore, the ubiquitous involvement of the visual system in MS renders the eye to provide readily accessible and non-invasive disease biomarkers. For example, electroretinography (ERG) and optical coherence tomography (OCT) are gaining attention as non-invasive tools for assessing retinal function and structure, respectively (5, 6). These measures correlate with neuroaxonal damage and have been proposed as potential markers for disability progression in both relapse (RMS) and secondary progressive MS (SPMS). However, variability in longitudinal measurements limits their utility at the individual level of people with MS (pwMS), necessitating further refinement of these techniques (7).

Another promising avenue lies in cellular markers derived from immune phenotyping. Dysregulated immune cell populations, including T-cells, B-cells, and myeloid cells, have been implicated in MS progression. The interplay between these cells not only drives inflammation but also contributes to central nervous system (CNS)-compartmentalized pathology seen in progressive forms of the disease. Understanding these cellular dynamics could pave the way for novel therapeutic strategies targeting specific immune pathways (8).

Despite these advances, the conversion from RMS to SPMS remains challenging to pinpoint clinically. This transition is characterized by a shift from relapse-driven disability to insidious progression, often without overt inflammatory activity. The combination of sNfL and GFAP levels has shown potential to improve diagnostic accuracy. However, determining the exact timing of this transition remains elusive in routine clinical practice (3, 4).

This study aims to address this unmet need by integrating inflammatory and neurodegenerative biomarkers to develop a Classification And Regression Tree (CART) model for identifying the transition from RMS to SPMS. By analyzing samples from pwMS affected by SPMS, RMS, and healthy controls, we seek to combine ophthalmological, neurodegenerative and cellular markers into a comprehensive diagnostic framework. This approach has the potential to optimize therapeutic strategies by enabling timely identification of disease progression and tailoring treatments accordingly.

## Methods

### Study design

This observational study was conducted between late 2021 and beginning of 2022 following approval by the Ethics Committee of the Otto-von-Guericke University Hospital Magdeburg (June 2021; registration number 73/21). A total of 52 participants were screened for eligibility, of which 45 were successfully recruited based on inclusion criteria. All included patients had a confirmed diagnosis of multiple sclerosis according to the 2017 McDonald criteria at the time of study inclusion. The study included pwMS affected by RMS, SPMS, and an age- and sex-matched healthy control (HC) group (Table 1). An interdisciplinary approach was employed, incorporating assessments of ophthalmological parameters—such as optical coherence tomography (OCT) and multifocal electroretinography (mfERG)—as well as neuro-immunological profiling through deep immunophenotyping and multiplex analysis (for specific marker values see Supplementary Table 1). Disease course classification was based on established clinical MS phenotype definitions. RMS was defined as a relapsing disease course, and SPMS as a secondary progressive disease course following an initial relapsing phase with continuous disability accumulation independent of relapses. In this study, SPMS classification additionally required evidence of insidious disability progression independent of relapses over a period of at least 12 months, including documented EDSS worsening, and the absence of clinical relapses or new inflammatory MRI activity during this interval. Phenotype assignment was based on the treating neurologists’ clinical classification at the time of study inclusion (9). Phenotype classification was not reassessed *post hoc* for study purposes but reflected the treating neurologists’ real-world clinical judgment based on the documented longitudinal disease course. These evaluations aimed to identify potential surrogate markers for MS disease progression. Additionally, computational CART analysis was performed to explore a stratification method capable of distinguishing between RMS and SPMS.

**Table 1:** Demographical characteristics of participating individuals.

Characteristics	SPMS	RMS	All MS	HC
Number of individuals: n	9	20	29	16
Female sex: *n* (%)	6 (66.6%)	13 (65.0%)	19 (65.5%)	10 (62.5%)
Age: years, mean ± SD	61.8 (±6.6)	42.8 (±11.6)	48.7 (±13.6)	44.6 (±14.2)
Disease duration: years, mean ± SD	24.1 (±5.6)	12.9 (±9.1)	16.4 (±9.7)	-
Expanded disability status scale (EDSS): mean ± SD	5.8 (±1.1)	2.7 (±1.3)	3.6(±1.9)	-
BMI: mean ± SD	25.2 (±5.4)	25.8 (±5.2)	25.6 (±5.3)	25.5 (±3.2)
MS therapy				
Siponimod (Mayzent)	3 (33.3%)	1 (5.0%)	4 (13.6%)	-
Cladribin (Mavenclad)	0 (0.0%)	1 (5.0%)	1 (3.4%)	-
DMF (Tecfidera)	1 (11.1%)	1 (5.0%)	2 (6.8%)	-
Fingolimod (Gilenya)	0 (0.0%)	5 (25.0%)	5 (17.0%)	-
Copaxone (Glatirameracetat)	0 (0.0%)	1 (5.0%)	1 (3.4%)	-
Natalizumab (Tysabri)	1 (11.1%)	8 (40.0%)	9 (30.6%)	-
Ocrevus (Ocrelizumab)	1 (11.1%)	1 (5.0%)	2 (6.8%)	-
Ozanimod (Zeposia)	0 (0.0%)	1 (5.0%)	1 (3.4%)	-
None	3 (33.3%)	1 (5.0%)	4 (13.6%)	

### Ophthalmological screening and examination

A complete standard eye examination was carried out to rule out abnormalities or diseases that might affect visual function except for MS related changes in the MS group. This included: (i) Visual acuity test to determine far (4 m) and near (~40 cm) best corrected visual acuity (BCVA) using the Early Treatment Diabetic Retinopathy Study chart (ETDRS chart); (ii) slit-lamp examination for anterior and posterior eye segments and intraocular pressure; (iii) visual field (VF) test with the Swedish Interactive Threshold Algorithm 24–2 protocol (SITA-Fast) of the Humphrey Field Analyzer 3 (Carl Zeiss Meditec AG, Jena, Germany). Exclusion criteria included: (i) BCVA > 0.2 (logarithm of minimum angle of resolution [logMAR]); (ii) VF defects or eye diseases affecting visual function; (iii) refractive errors > ± 5 Diopters.

For ophthalmological characterizations and correlations with neuroinflammatory/−degenerative mediators, only one eye per participant (*n* = 45 eyes) was randomly selected and included in the analysis as follows.

### Functional retinal assessment

Retinal function was assessed using multifocal electroretinography (mfERG) recordings, namely multifocal Photopic Negative Response of ERG (PhNR) according to Al-Nosairy et al. (6, 10, 11). We used VERIS Science (VERIS 5.1.12XScience; EDI: Electro-Diagnostic Imaging, Redwood City, CA, United States) and a monochrome CRT-monitor (MDG403, Philips; P45 phosphor) with 75 Hz frame rate for stimulation and recording of responses viewed binocularly at 36 cm distance. Responses were recorded with skin electrodes (12), a gold-cup skin electrode (10 mm diameter Golden EEG Cup Electrodes, Natus Manufacturing Limited, Ireland), placed on the lower lid 5 mm below the lid margin. The stimulus covered ±23° of the visual field, divided into five separate visual field locations, a central ring (0.0–4.8°) and 4 outer quadrants (4.8–23.1°). Stimulation followed an m-sequence of 2^9^–1 elements. Two stimulus states could be taken, “flash” (duration: 1 frame) and “no-flash” (duration: 8 frames). Mean luminance was 104 cd/m^2^ [min: 7 cd/m^2^ (“no-flash”); maxi: of 200 cd/m^2^ (“flash”)]. The recordings comprised 16 6.8-s segments and were performed 3 times with dilated pupils. The signals were amplified by 100 K (Grass Model 12, Astro-Med, Inc., West Warwick, RI, United States), band pass filtered 3–300 Hz and digitized at 1200 Hz. Traces were off-line digitally filtered (3–45 Hz) with Igor analysis software (IGOR Pro, WaveMetrics, Portland, United States). The traces from right eyes were left–right flipped, to depict visual fields of mfERG from both eyes. Following our previous results, the responses were grouped across all visual field locations stimulated and the following variables were extracted from mfERG for correlation with neuro-mediators: (i) amplitudes and peak times of the 1st negativity (N1), 1st positivity (P1), 2nd negativity (N2: Photopic negative response [PhNR]) and (ii) PhNR ratio of N2 to P1 amplitudes (11, 13). N1, P1, N2/PhNR-ratio indicate the function of cone-photoreceptors and bipolars, cone bipolars and horizontal cells, and retinal ganglion cells, respectively (14).

### Structural retinal assessment

Structural assessment of the retina for the (i) optic disc and (ii) macula was performed using a spectral domain OCT device (Heidelberg Spectralis^®^, Heidelberg Engineering, Heidelberg, Germany). OCT scans meeting the minimum quality score of ≥ 15 were included.

(i) Optic disc: the peripapillary retinal nerve fiber layer thickness (pRNFL) was assessed from a 3.5 mm (12°) circle scan. The pRNFL thickness was further calculated for different sectors, namely average (pRNFL_G), nasal (pRNFL_N), temporal (pRNFL_T) nasal/temporal ratio (pRNFL_N_T) and papillomacular bundle (pRNFL_PMB).
(ii) Macula: the structure of the macula was assessed covering an angle of 30° x 25° and using 61 vertical B scans (each with 768 A-Scans, automatic real-time = 13 frames). The combined ganglion cell layer and inner plexiform layer thickness (GCIPL) was calculated from the central (1 mm), paracentral (3 mm) and pericentral (6 mm) ETDRS circle protocol and averaged within 6 mm (GCIPL_G).

### Immunophenotyping

Blood sample collection was performed at the Department of Neurology of the University Clinic Magdeburg. Fresh venous blood was drawn in EDTA and serum coagulation tubes (Kabe Labor Technik, Nümbrecht, Germany). Peripheral blood mononuclear cells (PBMCs) from whole blood of healthy volunteers and pwMS were isolated by Ficoll Paque PLUS (GE Healthcare, Chicago, US) gradient centrifugation. Harvested PBMCs were frozen at 1° per min until final storage on −80 °C in CTL Cryo^™^ ABC Media (Immunospot, Cleveland, US) for subsequent immunophenotyping.

For in-depth immunophenotyping archived PBMCs were quickly thawed in a 37 °C water bath before resuspension in ice-cold PBS (Gibco) and washed once. A total of 1 × 10^6^ cells were stained per panel. Cells were preincubated with human TrueStain FcX^™^ (BioLegend, San Diego, US) to block nonspecific binding. Details regarding staining panels are listed in Table 2, and gating strategies are listed in Table 3. Staining panels and gating were modified based on Monaco et al. (15). All phenotyping experiments were performed on a BD FACSCelesta^™^ with BVR configuration (BD, Heidelberg, Germany) with standardized application settings and analyzed by BD FACS DIVA v9 software (BD, Heidelberg, Germany).

**Table 2:** List of antibodies used for in-depth immunophenotyping.

Surface marker	Clone	Fluorochrome	Company	Cat-Nr.	Panel
CD3	UCHT1	FITC	BioLegend	300440	1,3,4
CD4	RPAT4	APC fire 750	BioLegend	300560	1,2
CXCR5	J25204	PE/Dazzle 594	BioLegend	356928	1
CD45RA	H100	BV605	BioLegend	304134	1,3
CCR7	G043H7	PerCP/Cy5.5	BioLegend	353220	1,3
CCR6	11A9	PE	BD	559562	1
CXCR3	G025H7	BV650	BioLegend	353730	1
CCR4	L2A1H4	BV421	BioLegend	359414	1
CD25	M-A251	BV785	BioLegend	356140	2,5
CD127	A019D5	FITC	BioLegend	351312	2
CD8	SK1	APC Fire 750	BioLegend	344746	3
CD161	HP3G10	BV421	BioLegend	339914	3
TCR Vα7.2	3C10	BV785	BioLegend	351722	3
TCR γ/δ	11F2	APC	Miltenyi	130–113-500	3
CD11c	B-LY6	APC	BD	559877	4
CD14	HCD14	PE/Dazzle 594	BioLegend	325634	4
CD16	3G8	APC Fire 750	BioLegend	302060	4
CD56	HCD56	PerCP/Cy5.5	BioLegend	318322	4
CD123	6H6	BV605	BioLegend	306026	4
HLA-DR	L243	BV785	BioLegend	307642	4
CD19	H1B19	FITC	BioLegend	302206	5
CD24	ML5	BV605	BioLegend	311124	5
IgD	1A6-2	PE/Dazzle 594	BioLegend	348240	5
CD27	323	BV421	BioLegend	302824	5
CD38	HIT2	APC	BioLegend	303510	5

**Table 3:** Gating strategies for in-depth immuno-phenotyping of immune cell subsets.

Cell type	Panel 1—Thelper cells			
CD4 T-cells	CD3 + CD4+			
Tfh		CXCR5+ CD45RA−		
Th17		Not CXCR5+ CD45RA−	CCR6+ CXCR3−	
Th1			CCR6− CXCR3+	
Th1/17			CCR6+ CXCR3+	
Th2			CCR6− CXCR3−	CCR4+ CD45RA−
CD4 term eff				CCR7+ CD45RA+
CD4 naive				CCR7− CD45RA+

Cell type	Panel 2 – Treg	
Treg	CD3+ CD4+	CD127 low CD25 high

Cell type	Panel 3 – cytotoxic T-cells + MAIT			
T-cells	CD3+	CD8+		
γδ-T-cells		TCR γδ+		
MAIT		TCR γδ-	CD161high Vα7.2+	
CD8 T-cells			Not CD161high Not Vα7.2+ CD8+	
CD8 term eff				CD45RA+ CCR7−
CD8 naive				CD45RA+ CCR7+
CD8 eff mem				CD45RA− CCR7−
CD8 cent mem				CD45RA− CCR7+

Cell type	Panel 4 – monocytes			
Class mono	CD3−	CD16− CD14high		
mDC		CD16− CD56−	HLA-DR+	CD11c+ CD123−
pDC				CD11c− CD123+
NK-cells		CD16+ and/or CD56+	CD56+ CD11c−/low CD14−	
Non class Mono			CD11c+ CD14low CD16+	
Interm Mono			CD11c+ CD14high CD16 low	

Cell type	Panel 5 – B-cells		
B-cells	CD19+		
Exhausted B-cells		CD27− IgD−	
Naive B-cells		CD27− IgD+	
Non switched mem B-cells		CD27+ IgD+	
Switched mem B-cells		CD27+ IgD−	Not CD38high
Plasmablasts			CD38 high
Breg		CD27+	CD24+ CD38+
Activated/IL12 prod. Breg			CD25+ CD38−

Adapted from Monaco et al. (15).

### Neurodegeneration/inflammation marker

Soluble factors for neurodegeneration/−inflammation were measured in serum by three different approaches in duplicates per test (triplicates for Quanterix Neurology 2-Plex). First, we used LEGENDplex^™^ technology kits, namely LEGENDplex^™^ Human Neuroinflammation Panel 1 (13-plex; Cat.No. 740796; BioLegend, San Diego, United States) and LEGENDplex^™^ Human Neurodegenerative Biomarker Panel 1 (5-plex; Cat.No. 741198; BioLegend, San Diego, United States) according to manufacturer’s protocol. Analysis was not possible for TGF-β1 and TNF-*α* (Neuroinflammation Panel) as well as for Tau, Aβ42, Aβ40 and sNfL (Neurodegenerative Biomarker Panel), because 95% of measured values were below detection limits. Secondly, we performed sandwich ELISA for explorative neurodegeneration markers, namely Cathepsin S (Human Total Cathepsin S DuoSet ELISA; Cat.No. DY1183; R&D Systems, Minneapolis, United States), CCL5 (Human CCL5/RANTES DuoSet ELISA; Cat.No. DY278-05; R&D Systems, Minneapolis, United States), and Osteoactivin/GPNMB (Human Osteoactivin/GPNMB DuoSet ELISA; Cat.No. DY2550; R&D Systems, Minneapolis, United States), according to manufacturer’s protocol. Because of the increasing relevance of the sNfL and GFAP for SPMS together with the insufficient data from LEGENDplex^™^ for sNfL, we decided to use the Quanterix Simoa^®^ technology for determination of both factors in serum. Therefore, we used the Neurology 2-Plex B (GFAP, NF-l) Assay kit (Quanterix Corp., Billerica, United States) according to manufacturer’s protocol.

### Computational analysis

For the purpose of exploring potentially informative stratification markers, we used simple statistical comparisons together with complementary machine-learning approaches to examine differences between pwMS SPMS and RMS. Given the limited cohort size, we first focused on straightforward analyses of individual parameters, such as comparing summary statistics between groups and reviewing simple plots, in order to identify markers that appeared potentially relevant. In a second exploratory step, we applied decision-tree and random-forest methods to assess whether some of these observed group differences could also be reflected in a basic classification framework. The decision tree was of particular interest because of its transparent and intuitive structure, which may facilitate interpretation and communication, while the random forest was included as a more robust complementary method for exploratory feature ranking. These machine-learning analyses were used only in an exploratory way, to examine their possible usefulness for future work, and not to establish a definitive predictive model. For illustrative purposes, we also established an exemplary flowchart summarizing the computational process together with a didactic real-world example in Supplementary Figure 1.

### Classification and regression trees (CART)

CARTs are a tool to visualize and illustrate the differences between pwMS affected by SPMS and RMS, providing separation values. CART construct binary decision trees based on a training set of predefined classes. The tree evaluates features to determine the best split, dividing the data into smaller sub-groups (16). Feature selection, a primary aim in our study, is crucial for building an effective decision tree. The selected feature is assessed based on its ability to distinguish between different classes using the concept of impurity.

There are two popular definitions for impurity: entropy and the Gini index (17). In the current work, we employed the Gini index as the splitting criterion. While both the Gini index and information gain provide roughly the same accuracy for classification trees (18), we chose the Gini index for our analysis. It is calculated using the following formula:


$$I_{G}(t)=\sum_{i=1}^{l}p_{i}(t)(1-p_{i}(t))$$

Here l is the number of the classes and p_i_ represents the proportion of the sample in each specific class. The Gini index measures the impurity of a node in a decision tree. A low Gini index shows that a node has mostly members from one class, making it more “pure.” In contrast, a high Gini index suggests a mixture of classes within the node, showing higher impurity. Decision tree algorithms use the Gini index to determine the optimal splitting criterion for nodes during the tree-building process, aiming to maximize the purity of downstream nodes.

The decision tree usually tends to grow many branches, which consequently can lead to overfitting. Two widely-used methods are employed to avoid this: using stop-splitting criteria to halt the growth, together with pruning to remove nodes, thereby achieving an optimal balance between accuracy and generalization (17). In the current work, due to the limited size of the study group, we set the stopping criterion after one split. After two splits, the SPMS and RMS groups were always fully separated due to the small dataset size. Allowing further splits would have likely increased the variance and led to overfitting, thereby increasing errors on unseen data. This means the tree uses the Gini index to identify the best feature for separating the two groups, executes the separation, and then stops.

### Random Forest

Random Forest is an ensemble learning algorithm commonly used for classification and regression tasks. It constructs multiple CARTs during training, each based on a random subset of the training data through bootstrapping. Moreover, at each node of these trees, a random subset of features is considered for determining the best split. During prediction, each tree independently provides an output, and for classification tasks, the final prediction is typically determined by a majority vote among the trees. This approach reduces overfitting, enhances robustness to noise, and provides insight into feature importance (19).

For this study, the hyperparameters used in the Random Forest classifier are n_estimators = 100 and max_depth = 3. Here, n-estimators specifies the number of CARTs in the forest, while max_depth controls the maximum depth of each individual CART. These parameters were chosen through a grid search, taking into account similar cases and the specifics of the study data.

In Random Forest the feature importance is determined by randomly shuffling the values of each feature and observing the resulting impact on the model’s performance, and then analyzing individual CARTs within the Random Forest to understand the contribution of each feature to their predictions (20).

In a single CART, the importance of the node j, which is n_j_ can be calculated with following formula:


$$n_{j}=W_{j}C_{j-}W_{\text{left}(j)}C_{\text{Left}(j)-}W_{\text{Right}(j)}C_{\text{Right}(j)}$$

W_j_ is the node, j reachability probability and C_j_ is the Gini impurity of each node. The importance of each feature, or measurement in our case, can be calculated as follows:


$$F(j)=\frac{n_{j}}{\sum_{i=1}^{m}n_{i}}$$

In the previous formula m is the total number of the nodes in the tree. We used Random Forest to identify the most important features, as it provides greater robustness compared to a single CART. For a Random Forest with k trees, the importance of each feature is calculated as follows:


$$\text{Feature Importance}\;(i)=\frac{\sum_{j=1}^{m}F(j)}{k}$$

The importance coefficient for a Random Forest always is between 0 and 1, with 1 showing the most critical feature. Importance is determined by factors such as the number of features and their influence on predictions.

### Repeated random holdout/Monte Carlo validation

To explore the potential usefulness of the machine-learning methods in this setting, we used a repeated random holdout/Monte Carlo validation approach. In each repetition, approximately 20–30% of the SPMS and RMS cases were randomly assigned to the test set, while the remaining cases were used to train the model. The trained model was then evaluated on the unseen test subset, and this procedure was repeated many times with different random splits. Performance was summarized using accuracy and F1-score.

Because of the limited sample size, this analysis was intended only as an exploratory internal assessment. The purpose was not to establish a definitive predictive model, but rather to examine the potential performance and possible future usefulness of these machine-learning methods in a larger and more rigorously validated setting.

### Software

For computational tasks the programming language and software Python v3.13.5 was used. It provides access to many libraries, including the Sklearn library for decision tree computation, and matplotlib for data visualization purposes. Additionally, we utilized the StratifiedKFold class from the sklearn.model_selection module for cross-validation purposes. Throughout the project, we developed, modified, and adjusted code segments to better align with our analytical framework and study goals, which are available upon reasonable request.

## Results

### Ophthalmological assessment

**Figure 1:**
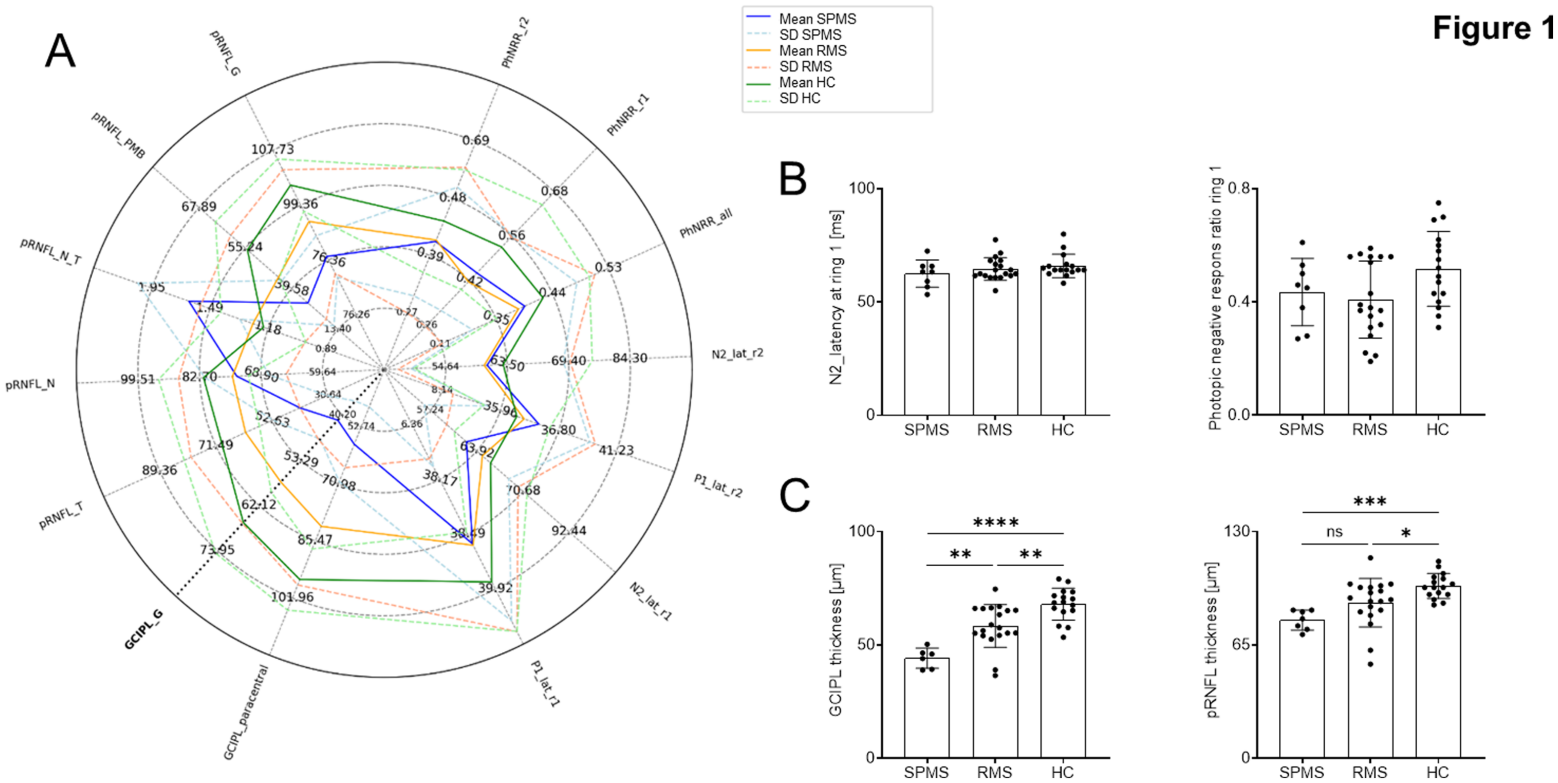
Ophthalmological assessment via functional mfERG readouts and OCT measurements shows significant decrease in structural integrity in pwMS in contrast to HC: **(A)** Spiderplot depicts the morphological parameters on the left and functional parameters on the right of all 3 groups based on ophthalmological assessment. Solid lines indicate the statistical mean of parameters, while dashed lines indicate the standard deviation. Functional assessment: These included measurements of P1 and Photopic negative response (PhNR) amplitudes, PhNR ratio as well as P1 and N2 peak times of the mfERG. These metrics were evaluated for the inner ring (r1), outer ring (r2) and the summed response across both rings (all). Structural assessment: Macular ganglion cell inner plexiform layers thickness (GCIPL) as a mean of the 6 mm ETDRS scan (GCIPL_G), and at paracentral (1–3 mm ETDRS scan). The peripapillary retinal fiber layer thickness (pRNFL) was also calculated for the mean value (pRNFL_G) and different sectors, namely Nasal (pRNFL_N), Temporal (pRNFL_T) and N/T ratio (pRNFL_N_T) as well as for the papillomacular bundle (pRNFL_PMB). **(B)** Most relevant functional parameters, PhNR ratio at ring 1 (PhNRR_r1) and N2 peak time (latency) at ring 1 (N2_lat_r1), were selected for specific inter group analysis. Retinal function was comparable across all groups. **(C)** Mean GCIPL was significantly different between all groups, while pRNFL thickness only differs between MS groups and HC. For B and C, dots indicate individually measured values, bars indicate the mean, while error lines show standard deviations. SPMS (secondary progressive multiple sclerosis); RMS (relapsing multiple sclerosis); HC (healthy controls); GCIPL (ganglion cell–inner plexiform layer); pRNFL (peripapillary retinal nerve fiber layer); after verifying normal distribution of data ordinary on-way ANOVA with Bonferroni correction for multiple comparisons was used for statistical tests; * *p* < 0.05; ** *p* < 0.01; *** *p* < 0.001; **** *p* < 0.0001.

To include both, functional and structural readouts from the retina and to ensure an unbiased, holistic approach, we included mfERG together with OCT measurements within our study. Interestingly, we could not detect any significant differences between HC, RMS and SPMS groups within the functional compartment, although central responses (PhNR_r1) indicated a trend of reduced responses in RMS vs. HC, but not SPMS vs. HC (Figures 1A,B). For structural results, we detected significantly thinner GCIPL in RMS than HC (*p* = 0.0011) and even greater differences between SPMS and HC (*p* < 0.0001). Additionally, we observed significantly thinner GCIPL in pwMS affected by SPMS rather than RMS, which indicates higher layer degeneration in the progressive form. SPMS participants without a history of ON still showed significant lower GCIPL than RMS participants without ON (45.25 μm vs. 63.56 μm, *p* = 0.004) -confirming that GCIPL thinning in the SPMS cohort was primarily driven by progressive neuroaxonal loss rather than residual damage from prior neuritis. The pRNFL thickness was also thinner in both MS groups compared to HC, but not altered between MS groups (Figure 1C). Furthermore, we found no significant differences in pRNFL thickness between pwMS affected by RMS and SPMS, regardless of optic neuritis (ON) history (*p* > 0.05 for all pairwise comparisons). This suggests that focal inflammatory damage (ON) and chronic neurodegeneration (SPMS) result in a similar degree of axonal loss in the peripapillary region, making pRNFL a less specific marker for distinguishing MS disease stages compared to GCIPL in this cohort (see Supplementary Table 2).

### Immunophenotyping

**Figure 2:**
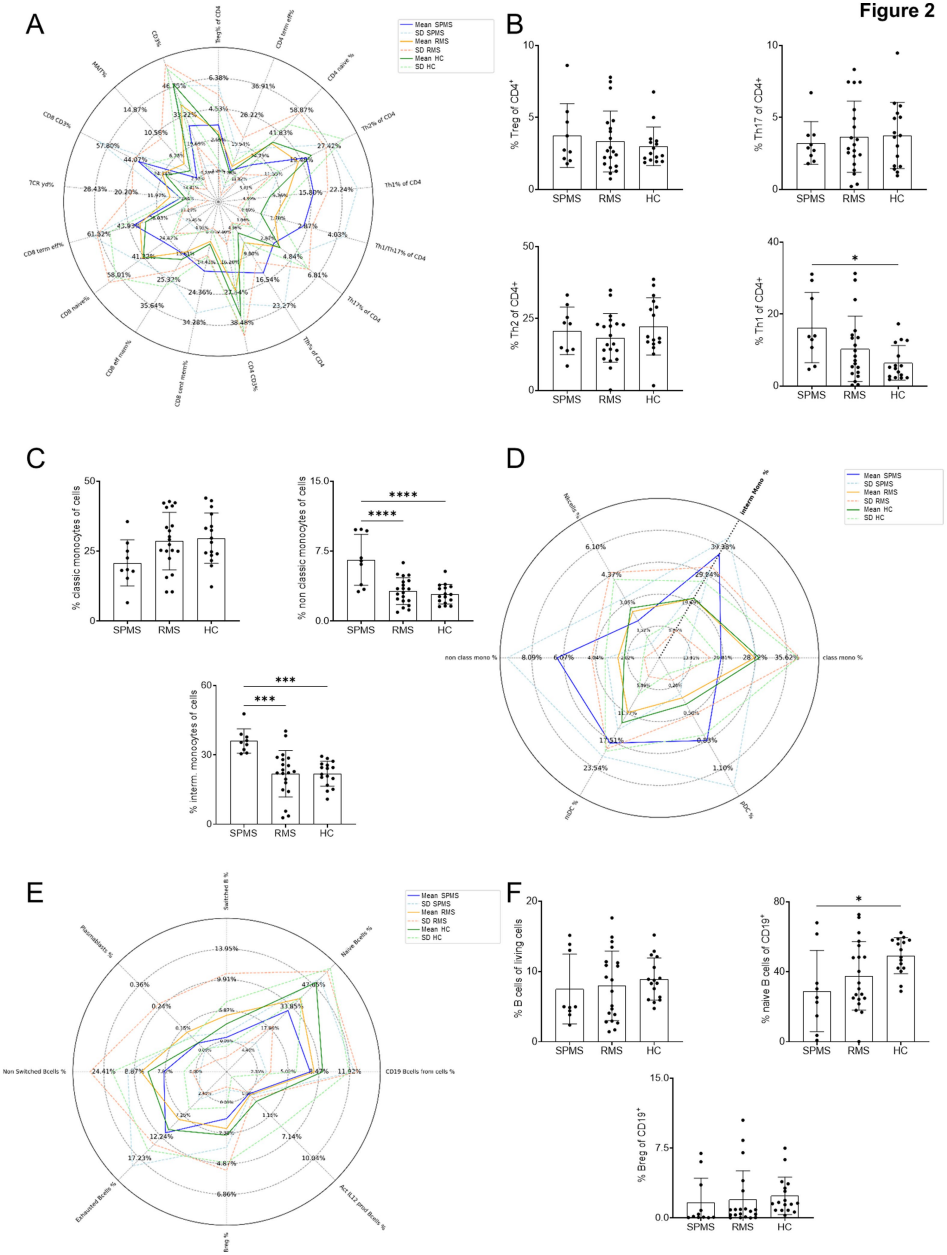
Immunophenotyping of peripheral blood from pwMS and HC. **(A)** Spider plot of all T-cell subsets. **(B)** Comparison of Tregs, Th17, Th2, and Th1 cells. **(C)** Non-classic and intermediate monocytes. **(D)** Spider plot of all monocyte subsets. **(E)** Spider plot of all B-cell subsets. **(F)** Comparison of total, naïve and regulatory B-cells. For all spider plots: Solid lines indicate the statistical mean of parameters, while dashed lines indicate the standard deviation. After verifying normal distribution of data ordinary on-way ANOVA with Bonferroni correction for multiple comparisons was used for statistical tests; * *p* < 0.05; ** *p* < 0.01; *** *p* < 0.001; **** *p* < 0.0001.

Extended immunophenotyping was performed to investigate cellular differentiation between disease entities. Analysis of T-cell subgroups revealed significantly higher Th1 cells in pwMS affected by SPMS compared to HC (Figures 2A,B). However, the most pronounced differences were observed in intermediate and non-classic monocytes. Both intermediate and non-classic monocyte populations were significantly elevated in the SPMS group compared to HC and RMS group. Figures 2C,D illustrate these findings, highlighting the distinct immunological profile of pwMS affected by SPMS. When comparing SPMS vs. RMS, while both MS subtypes showed alterations in monocyte subsets, pwMS affected by SPMS demonstrated significantly higher levels of intermediate and non-classic monocytes compared to the RMS group. In the RMS vs. HC comparison, pwMS affected by RMS showed moderate elevations in monocyte subsets compared to HC, but these differences were less pronounced than those observed in pwMS affected by SPMS. By investigating B-cell-subsets we could detect a significantly decreased number of naïve B-cells in the SPMS group compared to healthy controls (Figures 2E,F). In the RMS group these B-cells tended to be reduced but not statistically significant.

These findings suggest that quantification of intermediate and non-classic monocytes, in conjunction with Th1 cell levels and reduced numbers of naïve B-cells, may serve as potential biomarkers for distinguishing between SPMS, RMS, and healthy individuals. The observed cellular profile in pwMS affected by SPMS aligns with the hypothesis of enhanced inflammatory processes and altered immune regulation in this disease subtype (Figure 2).

### Markers of neurodegeneration

**Figure 3:**
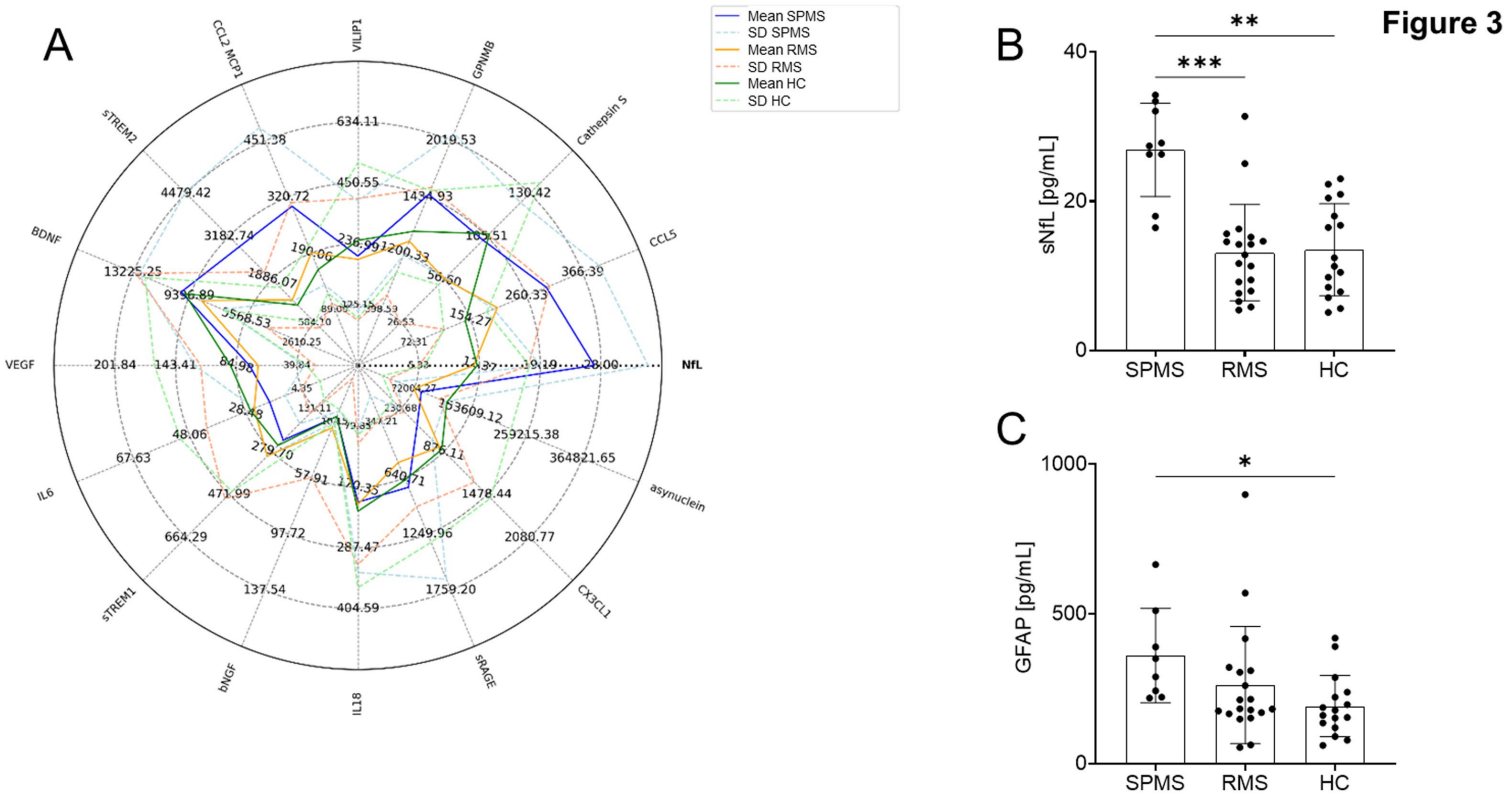
Neurodegenerative markers in peripheral blood of pwMS and HC. **(A)** Spider plot of all neurodegeneration markers. Solid lines indicate the statistical mean of parameters, while dashed lines indicate the standard deviation. **(B)** sNfL and **(C)**. GFAP concentration in all three groups measured by Quanterix Simoa^®^ technology. After verifying normal distribution of data ordinary on-way ANOVA with Bonferroni correction for multiple comparisons was used for statistical tests; * *p* < 0.05; ** *p* < 0.01; *** *p* < 0.001.

Analysis of neurodegenerative markers using LEGENDplex^™^ technology, ELISA and Quanterix Simoa^®^ technology only revealed significantly elevated levels of both sNfL and GFAP measured via Simoa^®^ in pwMS compared to HC. All other serum factors did not display any differences between SPMS, RMS or HC (Figure 3A). sNfL demonstrated a clear differentiation between pwMS groups. Specifically, sNfL levels were significantly higher in pwMS affected by SPMS compared to both HC (*p* < 0.01) and the RMS group (*p* < 0.001). This distinct elevation of sNfL in SPMS allows for a clear delineation of this group from both HC and RMS cohorts (Figure 3B). In contrast, GFAP showed a more limited differentiation capability. While GFAP levels were significantly elevated in pwMS affected by SPMS compared to HC (p < 0.01), there was no statistically significant difference observed between RMS and HC. Furthermore, GFAP levels did not significantly differ between pwMS affected by SPMS and RMS, limiting its utility in distinguishing between these two MS subtypes (Figure 3C).

These findings suggest that while both sNfL and GFAP are valuable markers of neurodegeneration in MS, sNfL demonstrates superior capability in distinguishing between HC, RMS, and SPMS groups. This enhanced discriminatory power of sNfL may provide a more sensitive tool for monitoring disease progression and potentially predicting conversion from RMS to SPMS.

### Computational analysis

Computational analysis was employed as an exploratory complement to identify and visualize potentially relevant features distinguishing SPMS from RMS. From over 100 measured parameters spanning clinical, ophthalmological, immunophenotyping, and neurodegeneration domains, 97 features with complete datasets were selected for downstream analysis. These parameters were examined in separate domains rather than as one single combined predictor set, and when variables were highly correlated or biologically very closely related, one representative feature was preferentially selected to reduce redundancy. Decision-tree and random-forest approaches were then applied as exploratory methods, and their performance was assessed by repeated random holdout/Monte Carlo validation. The resulting domain-specific feature rankings and mean validation accuracies are summarized in Table 4. Within this framework, intermediate monocytes, sNfL, and GCIPL_Gthickness showed relatively high average accuracy and F1-scores, around 80%, suggesting comparatively stronger discriminatory potential between RMS and SPMS. However, these findings need to be interpreted only as preliminary and as potentially promising for future studies, not as evidence of a definitive predictive model.

**Table 4:** Average accuracy and feature importance of identified parameters by computational analysis.

Test	Immunophenotyping		Neurodegeneration		OCT	
	Feature	Coefficient	Feature	Coefficient	Feature	Coefficient
Feature importance	Interm Mono	0.103	sNfL	0.176	GCIPL_G	0.238
	mDC	0.064	sTREM2	0.064	pRNFL_N_T	0.166
	Non class mono	0.052	Non class mono	0.052	GCIPL_paracentral	0.162
Mean Validation Accuracy	0.76		0.83		0.88	

**Figure 4:**
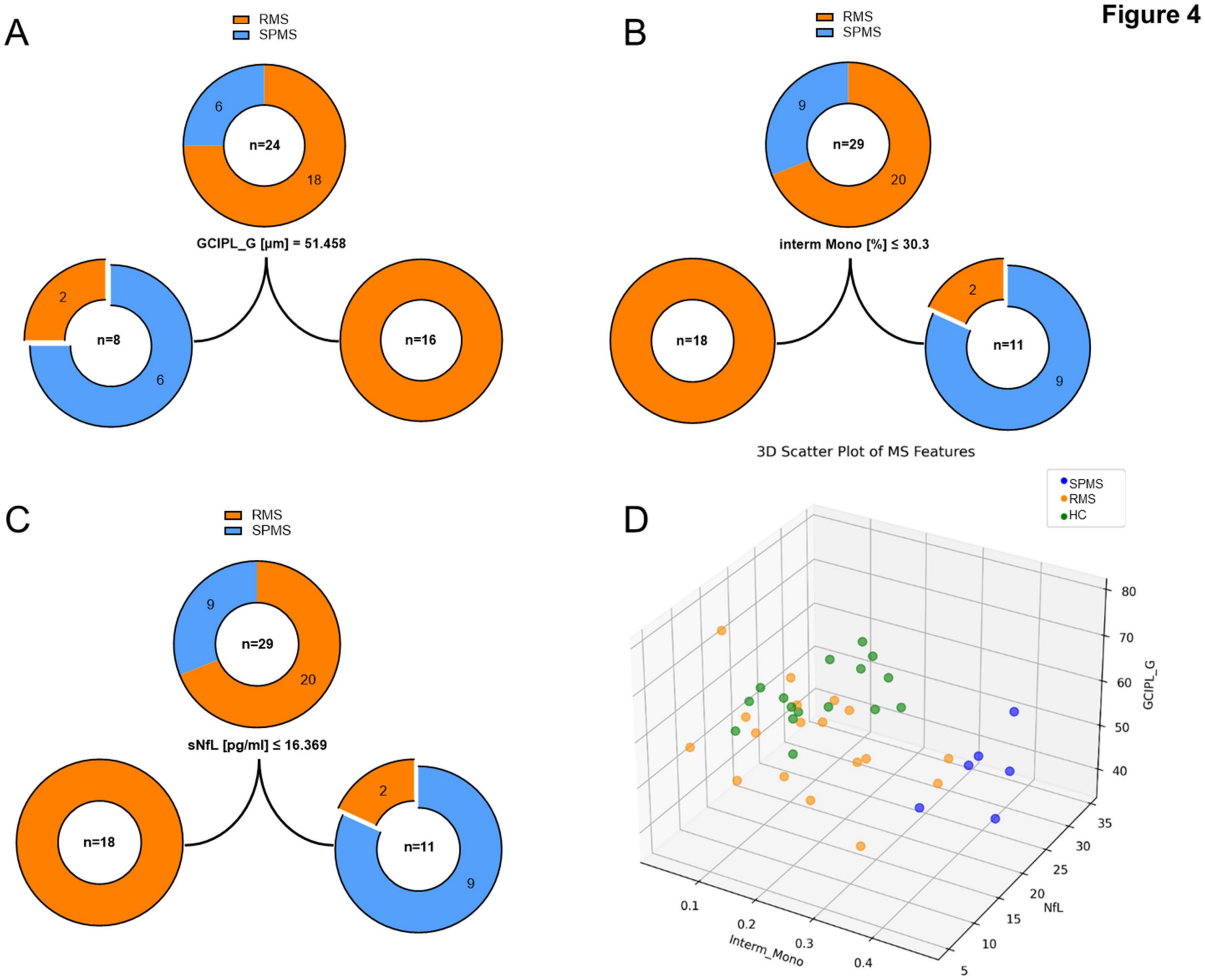
Random Forest analysis identified GCIPL thickness, intermediate monocytes and sNfL as best separators between RMS and SPMS. **(A–C)**. Visualization via single step CART of the 3 parameters with the best accuracy (80%) identified by Random Forest analysis. Upper circle depicts the overall group of pwMS consisting of RMS (orange) and SPMS (blue) (total *n* numbers in circles, number per group in sectors). Indicated below the middle circle are the related feature and the separation threshold, which separates the overall group into left (below or equal to threshold) and right (above threshold) groups. The best separating features are GCIPL thickness **(A)**, intermediate monocytes **(B)**, and sNfL **(C)**. As additional visualization we designed a three-dimensional representation of the identified parameters (**D**, Intermediate monocytes [% in decimals], sNfL [pg/ml] and GCIPL_G [μm]) together with a colored representation of RMS (orange) and SPMS (blue) and for visual comparison also HC (green).

To visualize these findings, we established a single CART with a max_depth of 1, resembling a one-step separation of RMS and SPMS groups by the three individually identified parameters (Figures 4A–C). The threshold of GCIPL thickness to distinguish between pwMS affected by RMS and SPMS was calculated as 51.458 μm. PwMS with thicker GCIPL were assigned into the RMS group, while pwMS with lower thickness were assigned to the SPMS group. During the separation two pwMS were misclassified as SPMS, while clinically were identified as pwMS affected by RMS (Figure 4A). CART for intermediate monocytes as well as for sNfL displayed similar results: thresholds for intermediate monocytes and sNfL were calculated as 30.3% from all lymphocytes and 16.369 pg/mL respectively, where lower results were classified as RMS and higher percentages were assigned as SPMS. Also 2 pwMS were misclassified as SPMS based on intermediate monocyte/sNfL percentages, while clinically being interpreted as RMS (Figures 4B,C). To further illustrate the three identified features among our study cohort, we established a three-dimensional scatter plot including not only pwMS affected by RMS and SPMS with all three parameters available, but also HC as comparison/benchmark (Figure 4D). Visually we could distinguish SPMS from HC very easily, as both groups cluster very differently. Observing the distribution of pwMS affected by RMS, three pwMS are strongly aligned with the SPMS cluster. Interestingly, we found these three pwMS consistently represented within the false categorized RMS group of the final CART results (Figures 4A–C).

## Discussion

Here we report that the combination of sNfL, intermediate monocytes, and GCIPL rather than as three separate biomarkers have the potential to enhance diagnostic certainty and serve as an adjunctive diagnostic tool in borderline cases to detect the transition from RMS to SPMS. The three biomarkers differentiate between pwMS with SPMS and RMS with an accuracy of 80%. This finding represents a step forward in our ability to distinguish between the two entities in MS, potentially leading to more tailored treatment approaches.

Our ophthalmological readouts confirm previously established differences, such as decreased RNFL thickness in pwMS, with a more pronounced reduction in SPMS cases. Additionally, we observed a decrease in GCIPL thickness, which was also more prominent in pwMS affected by SPMS. These findings align with current literature suggesting that GCIPL thickness correlates with disease progression in MS (21, 22). The progressive pRNFL thinning observed in our study is consistent with previous findings reporting increased pRNFL thinning over time, with average losses of 2.9 μm at 2 to 3 years and 6.1 μm at 3 to 4.5 years (21). This pattern was observed in eyes both with and without a prior history of optic neuritis (ON), supporting the notion of subclinical axonal loss in the anterior visual pathway in MS.

However, it is important to note that our results showed some discrepancies with previous studies. For instance, while it was reported a median pRNFL thickness reduction of 3.5 μm for ON eyes and 4.7 μm for eyes without ON over a five-year period (23, 24), the differences in our findings could be attributed to variations in study populations, including factors such as age, gender distribution, and disease duration. These discrepancies underscore the need for larger, more comprehensive studies to establish standardized norms for OCT measurements in pwMS.

Regarding neurodegenerative markers from peripheral blood, our results showed higher concentrations of sNfL together with GFAP in pwMS compared to HC. This observation supports the potential use of these markers as surrogate indicators of disease activity and progression, as suggested by previous literature (25). The elevated levels of sNfL in particular align with recent developments in sensitive assays for neurofilament light chain, which have made it a promising biomarker for predicting MS disease activity and progression (25).

However, it is crucial to acknowledge the limitations of using sNfL as a single marker. As pointed out by recent studies, sNfL levels are elevated in other neurodegenerative disorders as well, i.e., ALS and can be confounded by factors such as age, body mass index, and blood volume (25). Furthermore, there is considerable overlap in the range of serum sNfL levels compared to healthy controls (26). These confounding factors highlight the importance of using sNfL in conjunction with other biomarkers, as our study suggests, to improve diagnostic and predictive accuracy (27, 28).

Our immunophenotyping results revealed a significant increase in Th1 cells in pwMS affected by SPMS compared to healthy controls. Interestingly, we did not observe significant differences in the relative amounts of Th2, Th17, and Tregs. These findings partially align with previous studies that have reported alterations in T-cell subsets in pwMS and support the concept that inflammatory T-cell dysregulation persists in progressive MS, although the relative contribution of Th1 cells should be interpreted within a broader network that also includes Th17-related and upstream pathogenic programs (29–31). However, the specific pattern we observed, with a predominant increase in Th1-cells, adds nuance to our understanding of the immunological landscape in SPMS (26, 28).

The elevated levels of non-classic and intermediate monocytes in pwMS affected by SPMS compared to both pwMS displaying RMS and healthy controls represent a novel finding. This observation suggests a potential role for these monocyte subsets in the progression from RMS to SPMS. However, it is important to note that previous studies have shown conflicting results regarding monocyte subsets in MS (32, 33). These discrepancies could be due to differences in patient cohorts, disease duration, or methodological variations in cell isolation and characterization techniques (28, 34).

The analysis using Random Forest and CARTs, which identified sNfL, GCIPL, and intermediate monocytes as the most accurate predictors (about 80% accuracy) for distinguishing between RMS and SPMS, shows promise for refining our diagnostic capabilities. This combination of markers spans different aspects of MS pathology—axonal damage (sNfL), neuronal loss (GCIPL), and inflammation (intermediate monocytes)—providing a more comprehensive assessment of disease state.

However, it is crucial to interpret these results with caution. While an 80% accuracy is promising, it still leaves room for misclassification in a significant number of cases. The performance of this model needs to be validated in larger, independent cohorts to ensure its generalizability across diverse MS populations. On the other hand, we could identify the mismatched pwMS from final CARTs as those affected by RMS, which cluster near the SPMS group in a three-dimensional scatter plot, which might be indicative for predictive conversion of RMS to SPMS in these cases. A clinical follow-up of mismatched pwMS, to ensure a clinically identified switch to SPMS in these cases, seems highly warranted and will be monitored in the future.

A key limitation of this study is its cross-sectional design, which precludes assessment of longitudinal disease progression. While the investigated biomarkers show associations with disease phenotype, the study design does not allow conclusions regarding their predictive value for disease transition, particularly from RMS to SPMS. Longitudinal studies are required to determine whether these markers can reliably capture or predict progression over time. In addition, the relatively small sample size limits the statistical robustness and generalizability of our findings. However, as an exploratory pilot study assessing the feasibility of integrating multimodal biomarkers within a CART-based framework, the results should be considered hypothesis-generating rather than confirmatory and require validation in larger, independent cohorts to establish reproducibility and broader applicability.

In conclusion, our findings suggest that a multimodal approach—integrating neurodegenerative, ophthalmological, and inflammatory biomarkers—may enhance diagnostic certainty in borderline cases between RMS and SPMS. This approach could potentially lead to earlier and more accurate diagnosis of SPMS, allowing for more timely and appropriate therapeutic interventions. However, larger cohorts are needed in future studies to establish a robust, multilevel CART model as well as close monitoring of mismatched cases in respect to possible conversion to a progredient phenotype of MS. Such studies should also aim to address the limitations of our current work, including potential confounding factors such as age-related changes, comorbidities (e.g., diabetes, hypertension), and medication effects, as well as the need for longitudinal data to assess the predictive value of these markers over time.

Moreover, future research should explore the biological mechanisms underlying the observed changes in these markers, particularly the role of intermediate monocytes in MS progression. Understanding these mechanisms could not only improve our diagnostic capabilities but also potentially reveal new therapeutic targets. As we continue to refine our understanding of MS pathology and progression, integrating multiple biomarkers may become an essential tool in personalized MS management, enabling more tailored treatment approaches and improved patient outcomes.

## Data Availability

The original contributions presented in the study are included in the article/Supplementary material, further inquiries can be directed to the corresponding author.
