# Supplementary material for: From inflammation to neurodegeneration: an exploratory pilot study of a diagnostic framework for progression in MS
Source: Front Neurol. 2026 May 20;17:1767921. doi: 10.3389/fneur.2026.1767921 (PMC13229691; doi:10.3389/fneur.2026.1767921)
Supplement: Supplementary file 1 [file Supplementary_file_1.DOCX]

**Supplementary Figure 1**

**
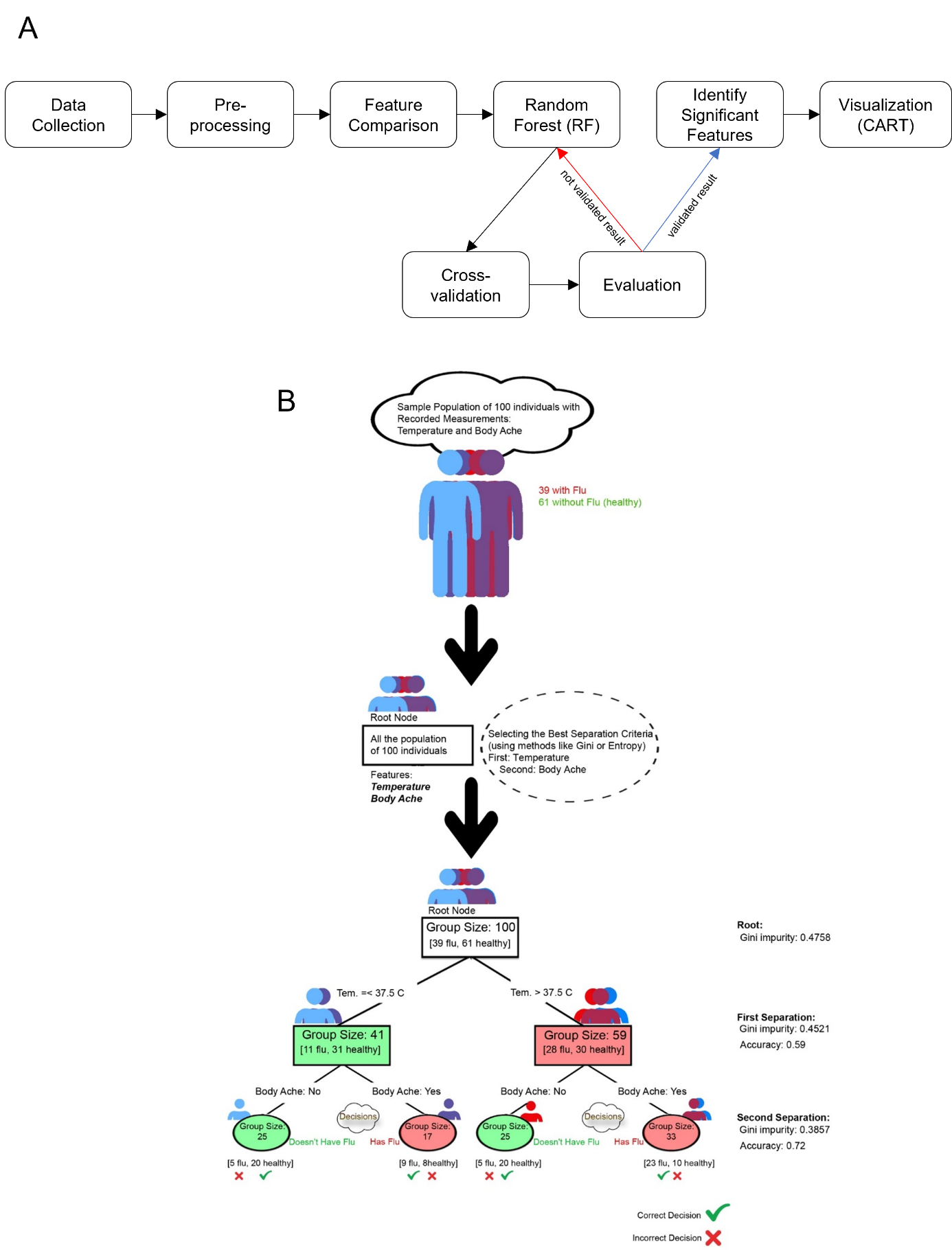
**

**Computational workflow for identification of significant features and schematic representation of a Decision Tree analysis.** A. Simplified workflow design shows the transition from raw, collected experimental data followed by preprocessing i.e. transposition into comparable data types and harmonization of data formats. All features were compared to identify the best features for separating RMS and SPMS groups via feature based, one step CARTs and subsequently transferred into Random Forest algorithm. If Crossvalidation and respective manual evaluation of the results were sufficient, significant features were identified and subsequently visualized via CART. B. Schematic representation of a two-dimensional decision tree used to diagnose influenza based on two independent variables: core body temperature and the presence of body pain. The first step seperates the initial study group of 100 individuals (39 with flu and 61 witout flu) into two groups based on an increased body temperature above 37.5 °C. This separation reached an accuracy of 59 %, based on the separation criteria. The second separation was done on the basis of the symptom of body aches, which led to 4 final groups: low body temperature without body pain, low body temperature with body pain, high body temperature without body pan and high body temperature without body pain. Based on the features (body ache and temperature) the algorithm subjected two of the final groups to “no flu” and two groups to “flu” which was calculated with an accuracy of 72%, which means 28 patients were computationally subjected to the wrong group, while 72 individuals were classified correctly.
