# Supplementary material for: From inflammation to neurodegeneration: an exploratory pilot study of a diagnostic framework for progression in MS
Source: Front Neurol. 2026 May 20;17:1767921. doi: 10.3389/fneur.2026.1767921 (PMC13229691; doi:10.3389/fneur.2026.1767921)
Supplement: Supplementary file 2 [file Table_1.DOCX]

Supplementary Table 1**. Additional Statistics for specific biomarkers of participating individuals**

| **Additional biomarker parameters: mean ± SD** | **SPMS** | **RMS** | **All MS** | **HC** | **Coefficient of variation (CV%)** |
| --- | --- | --- | --- | --- | --- |
| CD4 CD3% of lymphocytes | 18,99% (±12,82%) | 28,41% (±16,65%) | 25,49% (±15,96%) | 36,26% (±6,42%) | SPMS: 67.51% RMS: 58.61% All MS: 62.61% HC: 17.71% |
| Tfh% of CD4 | 13,42% (±9,33%) | 6,02% (±5,41%) | 8,31% (±7,54%) | 3,79% (±2,48%) | SPMS: 69.52% RMS: 89.87% All MS: 90.73% HC: 65.44% |
| Th17% of CD4 | 2,79% (±0,70%) | 4,00% (±2,78%) | 3,63% (±2,39%) | 3,37% (±1,77%) | SPMS: 25.09% RMS: 69.50% All MS: 65.84% HC: 52.52% |
| Th1/Th17% of CD4 | 2,44% (±1,82%) | 1,46% (±1,54%) | 1,76% (±1,66%) | 0,82% (±0,78%) | SPMS: 74.59% RMS: 105.48% All MS: 94.32% HC: 95.12% |
| Th1% of CD4 | 16,74% (±9,26%) | 10,48% (±9,10%) | 12,42% (±9,45%) | 6,02% (±4,63%) | SPMS: 55.32% RMS: 86.83% All MS: 76.09% HC: 76.91% |
| Th2% of CD4 | 22,67% (±6,98%) | 17,07% (±9,04%) | 18,81% (±8,73%) | 23,63% (±8,56%) | SPMS: 30.79% RMS: 52.96% All MS: 46.41% HC: 36.23% |
| CD4 naive% of CD4 | 19,85% (±12,00%) | 35,41% (±24,61%) | 30,58% (±22,49%) | 33,14% (±12,47%) | SPMS: 60.45% RMS: 69.50% All MS: 73.54% HC: 37.63% |
| CD4 term eff% of CD4 | 1,61% (±2,05%) | 5,50% (±11,37%) | 4,29% (±9,61%) | 3,02% (±4,32%) | SPMS: 127.33% RMS: 206.73% All MS: 224.01% HC: 143.05% |
| Treg% of CD4 | 3,70% (±2,24%) | 3,32% (±2,13%) | 3,44% (±2,13%) | 3,08% (±1,35%) | SPMS: 60.54% RMS: 64.16% All MS: 61.92% HC: 43.83% |
| CD3% of lymphocytes | 31,80% (±20,76%) | 38,61% (±18,41%) | 36,49% (±19,06%) | 50,06% (±6,55%) | SPMS: 65.28% RMS: 47.68% All MS: 52.23% HC: 13.08% |
| MAIT% of lymphocytes | 1,83% (±0,77%) | 4,38% (±4,93%) | 3,59% (±4,26%) | 2,89% (±2,86%) | SPMS: 42.08% RMS: 112.56% All MS: 118.66% HC: 98.96% |
| CD8 CD3% of lymphocytes | 37,21% (±17,02%) | 31,21% (±15,14%) | 33,07% (±15,69%) | 29,49% (±11,24%) | SPMS: 45.74% RMS: 48.51% All MS: 47.44% HC: 38.11% |
| TCR yd% of lymphocytes | 4,06% (±3,89%) | 7,21% (±10,90%) | 6,23% (±9,33%) | 2,75% (±1,54%) | SPMS: 95.81% RMS: 151.18% All MS: 149.76% HC: 56.00% |
| CD8 term eff% of CD8 | 38,90% (±24,18%) | 38,39% (±25,55%) | 38,55% (±24,70%) | 35,10% (±22,09%) | SPMS: 62.16% RMS: 66.55% All MS: 64.07% HC: 62.93% |
| CD8 naive% of CD8 | 32,40% (±17,26%) | 42,54% (±23,39%) | 39,39% (±21,89%) | 40,57% (±19,82%) | SPMS: 53.27% RMS: 54.98% All MS: 55.57% HC: 48.85% |
| CD8 eff mem% of CD8 | 11,73% (±4,96%) | 12,48% (±8,02%) | 12,25% (±7,13%) | 16,59% (±11,78%) | SPMS: 42.28% RMS: 64.26% All MS: 58.20% HC: 71.01% |
| CD8 cent mem% of CD8 | 16,97% (±14,12%) | 6,59% (±4,67%) | 9,81% (±9,78%) | 7,74% (±4,42%) | SPMS: 83.21% RMS: 70.86% All MS: 99.69% HC: 57.11% |
| CD19 B-cells% of lymphocytes | 8,61% (±5,28%) | 7,78% (±4,76%) | 8,04% (±4,85%) | 8,82% (±3,07%) | SPMS: 61.32% RMS: 61.18% All MS: 60.32% HC: 34.81% |
| Naive B-cells % of B-cells | 30,39% (±22,61%) | 39,21% (±19,85%) | 36,47% (±20,75%) | 48,79% (±10,42%) | SPMS: 74.40% RMS: 50.62% All MS: 56.90% HC: 21.36% |
| Switched B% of B-cells | 1,91% (±3,52%) | 3,73% (±6,06%) | 3,17% (±5,40%) | 2,44% (±3,29%) | SPMS: 184.29% RMS: 162.47% All MS: 170.35% HC: 134.84% |
| Plasmablasts% of B-cells | 0,06% (±0,06%) | 0,09% (±0,15%) | 0,08% (±0,13%) | 0,04% (±0,03%) | SPMS: 100.00% RMS: 166.67% All MS: 162.50% HC: 75.00% |
| Non Switched B-cells % of B-cells | 12,21% (±11,95%) | 13,60% (±12,39%) | 13,17% (±12,06%) | 12,20% (±9,41%) | SPMS: 97.87% RMS: 91.10% All MS: 91.57% HC: 77.13% |
| Exhausted B-cells% of B-cells | 10,44% (±7,71%) | 7,69% (±6,02%) | 8,54% (±6,58%) | 10,94% (±4,63%) | SPMS: 73.85% RMS: 78.28% All MS: 77.05% HC: 42.32% |
| Breg% of B-cells | 2,16% (±3,10%) | 1,72% (±2,71%) | 1,86% (±2,79%) | 2,41% (±2,09%) | SPMS: 143.52% RMS: 157.56% All MS: 150.00% HC: 86.72% |
| Act IL12 prod B-cells% of B-cells | 0,00% (±0,00%) | 0,01% (±0,01%) | 0,01% (±0,01%) | 0,03% (±0,09%) | SPMS: - RMS: 100.00% All MS: 100.00% HC: 300.00% |
| Class mono% of cells | 21,53% (±8,69%) | 28,77% (±10,33%) | 26,52% (±10,28%) | 29,38% (±9,24%) | SPMS: 40.36% RMS: 35.91% All MS: 38.76% HC: 31.45% |
| Interm Mono% of cells | 34,70% (±6,73%) | 21,90% (±10,09%) | 25,87% (±10,88%) | 21,70% (±5,53%) | SPMS: 19.39% RMS: 46.07% All MS: 42.06% HC: 25.48% |
| NK-cells% of cells | 2,78% (±1,00%) | 2,69% (±1,69%) | 2,72% (±1,49%) | 3,07% (±1,24%) | SPMS: 35.97% RMS: 62.83% All MS: 54.78% HC: 40.39% |
| Non class mono% of cells | 5,86% (±2,61%) | 3,13% (±1,48%) | 3,98% (±2,26%) | 2,96% (±1,03%) | SPMS: 44.54% RMS: 47.28% All MS: 56.78% HC: 34.80% |
| mDC% of cells | 16,84% (±3,38%) | 11,81% (±7,20%) | 13,37% (±6,64%) | 13,46% (±5,80%) | SPMS: 20.07% RMS: 60.97% All MS: 49.66% HC: 43.09% |
| pDC% of cells | 0,82% (±0,41%) | 0,44% (±0,15%) | 0,56% (±0,31%) | 0,52% (±0,28%) | SPMS: 50.00% RMS: 34.09% All MS: 55.36% HC: 53.85% |
| CCL5 | 295,3712 (±106,2996) | 201,3711 (±103,3925) | 230,5435 (±111,5398) | 143,0273 (±43,1462) | SPMS: 35.99% RMS: 51.34% All MS: 48.38% HC: 30.17% |
| Cathepsin S | 104,7866 (±26,8155) | 78,9048 (±33,5394) | 86,9371 (±33,4254) | 107,7567 (±37,0923) | SPMS: 25.59% RMS: 42.51% All MS: 38.45% HC: 34.42% |
| GPNMB | 1576,4873 (±448,4168) | 1305,1469 (±404,2448) | 1389,3560 (±429,7204) | 1294,8755 (±470,6653) | SPMS: 28.44% RMS: 30.97% All MS: 30.93% HC: 36.35% |
| VILIP1 | 141,7271 (±92,4781) | 214,8046 (±198,7206) | 192,1253 (±174,4248) | 245,1034 (±254,1924) | SPMS: 65.25% RMS: 92.51% All MS: 90.79% HC: 103.71% |
| CCL2 MCP1 | 256,4153 (±162,6806) | 204,1905 (±113,7121) | 220,3982 (±130,1547) | 172,5577 (±49,8255) | SPMS: 63.44% RMS: 55.69% All MS: 59.05% HC: 28.87% |
| sTREM2 | 2478,5852 (±1546,9347) | 1325,1035 (±837,9216) | 1683,0806 (±1206,2722) | 1199,7977 (±530,6003) | SPMS: 62.41% RMS: 63.23% All MS: 71.67% HC: 44.22% |
| BDNF | 9322,3228 (±2488,2311) | 9023,6392 (±3206,7458) | 9116,3341 (±2960,8471) | 9670,8802 (±2058,0955) | SPMS: 26.69% RMS: 35.54% All MS: 32.48% HC: 21.28% |
| VEGF | 53,3027 (±27,0231) | 69,6834 (±54,3977) | 64,5998 (±47,7084) | 88,4765 (±73,4260) | SPMS: 50.70% RMS: 78.06% All MS: 73.85% HC: 82.99% |
| IL6 | 15,7067 (±6,7034) | 25,0815 (±18,3300) | 22,1721 (±16,1342) | 22,5924 (±25,8422) | SPMS: 42.68% RMS: 73.08% All MS: 72.77% HC: 114.38% |
| sTREM1 | 231,5476 (±63,0541) | 336,3711 (±185,2517) | 303,8397 (±163,8873) | 267,8290 (±207,6038) | SPMS: 27.23% RMS: 55.07% All MS: 53.94% HC: 77.51% |
| bNGF | 6,0735 (±4,7489) | 17,2136 (±45,4030) | 13,7563 (±37,8521) | 6,2493 (±6,3679) | SPMS: 78.19% RMS: 263.76% All MS: 275.16% HC: 101.90% |
| IL18 | 186,4627 (±64,5229) | 218,1662 (±104,5157) | 208,3271 (±93,9398) | 240,1008 (±131,7948) | SPMS: 34.60% RMS: 47.91% All MS: 45.09% HC: 54.89% |
| sRAGE | 511,9637 (±576,1792) | 562,4515 (±401,0440) | 549,1652 (±436,4117) | 747,4472 (±626,6931) | SPMS: 112.54% RMS: 71.30% All MS: 79.47% HC: 83.84% |
| CX3CL1 | 377,3743 (±212,1505) | 790,5808 (±488,7536) | 652,8453 (±457,2540) | 794,6437 (±747,2968) | SPMS: 56.22% RMS: 61.82% All MS: 70.04% HC: 94.04% |
| α-synuclein | 27287,2355 (±15430,3905) | 32888,8149 (±48805,8967) | 31150,3937 (±41126,0385) | 97880,6377 (±131109,8856) | SPMS: 56.55% RMS: 148.40% All MS: 132.02% HC: 133.95% |
| sNfL | 26,0701 (±7,3223) | 12,5498 (±6,6182) | 16,8956 (±9,2970) | 14,0296 (±6,0011) | SPMS: 28.09% RMS: 52.74% All MS: 55.03% HC: 42.77% |
| P1_lat_r1 | 39,0476 (±1,2199) | 39,0352 (±1,2808) | 39,0385 (±1,2403) | 39,5001 (±0,7585) | SPMS: 3.12% RMS: 3.28% All MS: 3.18% HC: 1.92% |
| N2_lat_r1 | 61,0713 (±4,8042) | 64,7369 (±4,9383) | 63,7500 (±5,0840) | 66,0556 (±5,3310) | SPMS: 7.87% RMS: 7.63% All MS: 7.97% HC: 8.07% |
| P1_lat_r2 | 37,1427 (±1,0603) | 36,7105 (±1,4823) | 36,8269 (±1,3748) | 36,4444 (±0,6659) | SPMS: 2.85% RMS: 4.04% All MS: 3.73% HC: 1.83% |
| N2_lat_r2 | 61,4286 (±8,5488) | 61,0965 (±10,2178) | 61,1859 (±9,6298) | 61,0554 (±9,0439) | SPMS: 13.92% RMS: 16.72% All MS: 15.74% HC: 14.81% |
| PhNRR_all | 0,3858 (±0,0875) | 0,3915 (±0,1246) | 0,3899 (±0,1141) | 0,4214 (±0,0765) | SPMS: 22.68% RMS: 31.83% All MS: 29.26% HC: 18.15% |
| PhNRR_r1 | 0,4313 (±0,1264) | 0,4254 (±0,1569) | 0,4270 (±0,1469) | 0,5008 (±0,1214) | SPMS: 29.31% RMS: 36.88% All MS: 34.40% HC: 24.24% |
| PhNRR_r2 | 0,3953 (±0,0986) | 0,4156 (±0,1308) | 0,4101 (±0,1214) | 0,4456 (±0,0976) | SPMS: 24.94% RMS: 31.47% All MS: 29.60% HC: 21.90% |
| pRNFL_G | 78,3333 (±5,6095) | 89,4737 (±14,0490) | 86,8000 (±13,3479) | 98,8000 (±7,3892) | SPMS: 7.16% RMS: 15.70% All MS: 15.38% HC: 7.48% |
| pRNFL_PMB | 36,3333 (±6,3140) | 46,2632 (±12,7447) | 43,8800 (±12,2008) | 54,4286 (±9,0274) | SPMS: 17.38% RMS: 27.55% All MS: 27.80% HC: 16.59% |
| pRNFL_N_T | 1,6900 (±0,3295) | 1,2868 (±0,3155) | 1,3836 (±0,3580) | 1,1840 (±0,2491) | SPMS: 19.50% RMS: 24.52% All MS: 25.87% HC: 21.04% |
| pRNFL_N | 72,0000 (±8,0250) | 75,5789 (±14,2178) | 74,7200 (±12,9406) | 80,6667 (±10,9783) | SPMS: 11.15% RMS: 18.81% All MS: 17.32% HC: 13.61% |
| pRNFL_T | 43,8333 (±8,1097) | 62,0526 (±16,3281) | 57,6800 (±16,6351) | 69,8667 (±11,8132) | SPMS: 18.50% RMS: 26.31% All MS: 28.84% HC: 16.91% |
| GCIPL_G | 44,2000 (±4,9640) | 59,2500 (±9,8782) | 56,1146 (±10,9377) | 67,7611 (±7,2505) | SPMS: 11.23% RMS: 16.67% All MS: 19.49% HC: 10.70% |
| GCIPL_paracentral | 58,4000 (±11,3683) | 82,0132 (±16,5648) | 77,0938 (±18,2532) | 94,4500 (±8,0933) | SPMS: 19.47% RMS: 20.20% All MS: 23.68% HC: 8.57% |
