# Supplementary material for: From inflammation to neurodegeneration: an exploratory pilot study of a diagnostic framework for progression in MS
Source: Front Neurol. 2026 May 20;17:1767921. doi: 10.3389/fneur.2026.1767921 (PMC13229691; doi:10.3389/fneur.2026.1767921)
Supplement: Supplementary file 3 [file Table_2.DOCX]

Supplementary Table 2: Retinal Layer Thickness classified by Multiple Sclerosis Subtype and History of Optic Neuritis

| **Group** | **n** | **GCIPL-G Mean ± SD [μm]** | **pRNFL-G Mean ± SD [μm]** |
| --- | --- | --- | --- |
| - **HC** | 17 | 68.14 ± 6.87 | 98.82 ± 6.92 |
| **RMS** | 19 | 58.81 ± 9.47^1,3^ | 89.16 ± 13.87^1^ |
| - RMS+ON | 9 | 53.54 ± 10.06^1,2^ | 82.78 ± 15.56^1^ |
| - RMS-ON | 10 | 63.56 ± 6.05^1,2,4^ | 94.90 ± 9.59 |
| **SPMS** | 7 | 44.17 ± 4.44^1,3^ | 79.29 ± 5.71^a^ |
| - SPMS+ON | 3 | 43.08 ± 3.47^1,4^ | 78.33 ± 5.86^1^ |
| - SPMS-ON | 4* | 45.25 ± 5.81^1,4^ | 80.00 ± 6.38^1^ |
